# Supplementary material for: Disrupted mitochondrial function in the Opa3L122P mouse model for Costeff Syndrome impairs skeletal integrity
Source: Hum Mol Genet. 2016 Apr 22;25(12):2404–16. doi: 10.1093/hmg/ddw107 (PMC5181626; doi:10.1093/hmg/ddw107)
Supplement: Supplementary Data [file supp_25_12_2404__index.html]

Disrupted mitochondrial function in the Opa3L122P mouse model for Costeff Syndrome impairs skeletal integrity — Disrupted mitochondrial function in the Opa3L122P mouse model for Costeff Syndrome impairs skeletal integrity — Supplementary Data 

# Disrupted mitochondrial function in the Opa3L122P mouse model for Costeff Syndrome impairs skeletal integrity

## Supplementary Data

files

- Supplementary Data - doc file
