# Supplementary material for: Disrupted mitochondrial function in the Opa3L122P mouse model for Costeff Syndrome impairs skeletal integrity
Source: Hum Mol Genet. 2016 Apr 22;25(12):2404–16. doi: 10.1093/hmg/ddw107 (PMC5181626; doi:10.1093/hmg/ddw107)
Supplement: Supplementary Data [file supp_ddw107_P45-OPA3-mutant_skeletal_impairment_Highlighted_Revision.doc]

**Disrupted mitochondrial function in the Opa3^L122P^ mouse model for Costeff Syndrome impairs skeletal integrity**

Alice E Navein^1^, Esther J Cooke^1^, Jennifer R Davies^2^, Terence G Smith^2^, Lois HM Wells^1,3^, Atsushi Ohazama^4^, Christopher Healy^4^, Paul T Sharpe^4^, Sam L Evans^5^, Bronwen AJ Evans^6^, Marcela Votruba^2,7^, Timothy Wells^1^

^1^School of Biosciences, Cardiff University, Museum Avenue, Cardiff, CF10 3AX, UK

^2^School of Optometry and Vision Sciences, Cardiff University, Maindy Road, Cardiff, CF24 4LU, UK

^3^Caerleon Comprehensive School, Cold Bath Road, Caerleon, Newport, NP18 1NF, UK

^4^Department of Craniofacial Development and Stem Cell Biology, King’s College London, Guy’s Hospital, Guy’s Tower, Floor 27, London Bridge, London, SE1 9RT, UK

^5^School of Engineering, Cardiff University, The Parade, Cardiff, CF24 3AA, UK

^6^Institute of Molecular and Experimental Medicine, School of Medicine, Cardiff University, Heath Park, Cardiff, CF14 4XN, UK

^7^Cardiff Eye Unit, University Hospital of Wales, Heath Park, Cardiff, CF14 4XW, UK

***Author for correspondence:**

Dr Timothy Wells

School of Biosciences

Cardiff University

Museum Avenue

Cardiff

CF10 3AX

UK

Tel: +44 (0)2920 874977

Fax: +44 (0)2920 876328

E-mail: [wellst@cardiff.ac.uk](mailto:wellst@cardiff.ac.uk)

**Abstract**

Mitochondrial dysfunction connects metabolic disturbance with numerous pathologies, but the significance of mitochondrial activity in bone remains unclear. We have, therefore, characterized the skeletal phenotype in the Opa3^L122P^ mouse model for Costeff syndrome, in which a missense mutation of the mitochondrial membrane protein, Opa3, impairs mitochondrial activity resulting in visual and metabolic dysfunction. Although widely expressed in the developing normal mouse head, Opa3 expression was restricted after E14.5 to the retina, brain, teeth, and mandibular bone. Opa3 was also expressed in adult tibiae, including at the trabecular surfaces and in cortical osteocytes, epiphyseal chondrocytes, marrow adipocytes and mesenchymal stem cell rosettes. Opa3^L122P^ mice displayed craniofacial abnormalities, including undergrowth of the lower mandible, accompanied in some individuals by cranial asymmetry and incisor malocclusion. Opa3^L122P^ mice showed an 8-fold elevation in tibial marrow adiposity, due largely to increased adipogenesis. In addition, femoral length and cortical diameter and wall thickness were reduced, the weakening of the calcified tissue and the geometric component of strength reducing overall cortical strength in Opa3^L122P^ mice by 65%. In lumbar vertebrae reduced vertebral body area and wall thickness were accompanied by a proportionate reduction in marrow adiposity. Although the total biomechanical strength of lumbar vertebrae was reduced by 35%, the strength of the calcified tissue (σ_max_) was proportionate to a 38% increase in trabecular number. Thus, mitochondrial function is important for the development and maintenance of skeletal integrity, impaired bone growth and strength, particularly in limb bones, representing a significant new feature of the Costeff syndrome phenotype.

**Introduction**

The mitochondrion represents a sub-cellular powerstation, utilizing protons from dietary carbohydrates and fat to generate heat and adenosine triphosphate (ATP). This function is particularly vital in tissues with high energy demands, such as the central nervous system, muscle, brown adipose tissue (BAT) and the endocrine glands. As a consequence, defective mitochondrial function is emerging as a potentially important mechanistic link between metabolic disturbance and the development of a wide range of pathologies (1,2,3).

A number of lines of evidence suggest that mitochondrial function may also be important in bone growth and mineralization. For example, in the context of longitudinal growth, chondrocyte maturation in the epiphyseal plate is accompanied by increased mitochondrial number and decreased mitochondrial size (4). Once in the hypertrophic zone, the chondrocyte mitochondria regulate calcification (5), a decrease in mitochondrial membrane potential accompanying subsequent chondrocyte apoptosis (6,7). In the context of bone remodelling, mitochondria are also abundant in both osteoblasts (8) and osteoclasts (9). In the osteoblastic lineage increased mitochondrial activity regulates osteoblast differentiation (10,11), the subsequent development of mitochondrial granules in fetal bone representing the first mineral deposits in the osteoid (12,13). Indeed, increasing mitochondrial calcium uptake in osteoblasts is thought to mediate the parathyroid hormone (PTH)-induced reduction in mitochondrial membrane potential (14). In addition, the expression of genes associated with mitochondrial dysfunction are rapidly increased following mechanical loading (15), with extreme mechanical loading of osteoblastic cells inhibiting mitochondrial activity and enhancing reactive oxygen species production (16). Although mitochondrial biogenesis is co-ordinated with osteoclast development (17), little is known about the functional role of osteoclast mitochondria. Despite these strands of evidence, the overall effect of mitochondrial dysfunction on bone integrity remains poorly characterized.

To address this question, we have characterized the bone phenotype in a murine model of primary mitochondrial dysfunction, the B6; C3-*Opa3*^L122P^ mouse (hereafter referred to as the Opa3^L122P^ mouse; 18). The Optic atrophy 3 (*OPA3*) gene gives rise to the production of Opa3A (hereafter called Opa3), a 179aa peptide containing an N-terminal mitochondrial targeting sequence (19,20), which directs expression to either the inner (21) or outer (20,22) mitochondrial membrane where it induces mitochondrial fragmentation (20). Mutations in Opa3A lead to the development of the hereditary optic neuropathy known as type III methyglutaconic aciduria (3-MGA) or Costeff syndrome (MIM 258501) (19,23).

Homozygous Opa3^L122P^ mice display the characteristic Costeff phenotype of 3-MGA (24), reduced visual acuity, spasticity and extrapyramidal dysfunction (18). However, we have recently shown that disrupted mitochondrial morphology in Opa3^L122P^ mice (25) is accompanied by phenotypic abnormalities not previously reported in the human Costeff syndrome kindreds, impaired thermogenesis in the brown adipose tissue (BAT) resulting in profound intra-abdominal leanness and post-natal growth retardation (24).

In this study we have characterized Opa3 mRNA expression in the murine skeleton and examined the effect of the Opa3^L122P^ mutation on skeletal integrity, including the 3-dimensional morphology of the skull, tibial and vertebral marrow adiposity and the geometry, trabecular microarchitecture and mechanical strength of the femora and lumbar vertebrae. This study demonstrates the impact of mitochondrial dysfunction on skeletal integrity, uncovering a striking novel aspect of the Costeff syndrome phenotype.

**Results**

*Skeletal Opa3 expression*

*Opa3* was expressed ubiquitously in the developing head of WT mice until E13.5 (Fig 1A & B), but from E14.5 expression of *Opa3* became more restricted, being seen in the developing submandibular salivary glands at E14.5 (Fig 1Ci), the sensory layer of the retina and in hair follicles (Fig 1Cvii & Di) and the developing brain and mandibular bone, but not in Meckel’s cartilages (Fig 1Cii & iii), or in developing skull bone (Fig 1Dii). Expression was also observed in developing cap stage tooth germs at E14.5 (Fig 1Civ-vi), whilst in the molars, *Opa3* was strongly expressed in odontoblasts while it was weakly expressed in ameloblasts at E18.5 (Fig 1Diii). In the incisors the immature odontoblasts showed strong *Opa3* expression while *Opa3* was weakly expressed in mature odontoblasts at E18.5 (Fig 1Div-vii). In contrast, strong *Opa3* expression was observed in ameloblasts in both anterior and posterior regions of incisors at E18.5 (Fig 1Div-vii).

Immunohistochemistry (IHC) was used to show that the most pronounced Opa3 expression in adult tibiae was observed in the marrow cavity (Fig 2A). Discrete expression was observed in cells on the endosteal and trabecular surfaces (Fig 2C) and on the surfaces of the cortical periosteum (Fig 2E) and synovial cartilage (Fig 2G). In addition, Opa 3 immunoreactivity was seen in cortical osteocytes (Fig 2E) articular chondrocytes (Fig 2G) and in the germinal, proliferative and hypertrophic chondrocytes of the proximal epiphyseal plate (Fig 2I & K). In the marrow cavity, Opa 3 immunoreactivity was observed around the unilocular lipid droplets of the adipocytes (Fig 2I & K) and in distinct rosettes of mesenchymal stem cells in the mid-diaphyseal marrow (Fig 2M).

*Skull bone structure in Costeff syndrome mice*

No abnormalities were observed in heads of E18.5 Opa3^L122P^ mice (data not shown), but µ-CT analysis revealed an impairment in postnatal skull development. Overall skull length was reduced by 10% in Costeff syndrome mice at P14 (Table 1, Fig 3; *P*<0.001), which appears to be accompanied in adult mice by a shortening of the snout (Fig 3). An 8% reduction in nasal height in Costeff syndrome mice at P14 (Table 1; Fig 3; *P*<0.05) was accompanied by reductions of 11% and 14% in the height and length of the lower mandible (Table 1; Fig 3; *P*<0.01 and *P*<0.001 respectively). Individual Costeff syndrome mice displayed varying degrees of cranial asymmetry, which was particularly observable in the shape of the occipital bone and accompanied by misalignment of the lower mandible and incisors (Table 1; Fig 3; *P*<0.05). At P14 mean incisor length in Costeff syndrome mice was 86% of that in WT littermates, but this was only significantly different in the lower incisors (Table 1; Fig 3; *P*<0.05). However, malocclusion in adult Opa3^L122P^ mice (Fig 3) was accompanied in some individuals by incisor elongation.

*Marrow adiposity in Costeff syndrome mice*

Adiposity in the tibial marrow cavity of Opa3^L122P^ mice was increased 8-fold (Fig 4A; *P*<0.05), due largely to a 6-fold elevation in adipocyte number (*P*<0.05). This increase was especially prominent at the lower end of the size spectrum (Fig 4A), with mean size being unaffected (inset). In contrast, although overall marrow adiposity in the L3 vertebral body was unaffected (Fig 4B), the total number of marrow adipocytes was reduced in Opa3^L122P^ mice by 45% (*P*<0.05). This reduction was especially prominent for smaller adipocytes, with mean size unaffected (Fig 4B inset). It should be noted that since the cross sectional area of vertebral marrow compartment in Opa3^L122P^ mice was only 79% of that in W-T mice (*P*=0.055), this reduction in adipocyte number was proportionate to the size of the vertebral marrow compartment (data not shown).

*Femoral structure and strength in Costeff syndrome mice*

Opa3^L122P^ mice showed a 25% reduction in femoral length (Fig 5A; *P*<0.001), with outer cortical diameter at the site of strength testing reduced in Opa3^L122P^ mice by 13% (Fig 5A; *P*<0.05). Although mean inner diameter in Costeff syndrome mice was only 90% of that in W-T mice, this was not statistically significant (*P*=0.072), but mean wall thickness in the mid-diaphyseal cortex was reduced by 31% (Fig 5A; *P*<0.01).

A 33% reduction in trabecular volume of Costeff syndrome mice (BV; Fig 5B *P*<0.01), was in direct proportion to the 35% reduction in total volume (TV; *P*<0.01; data not shown), so that relative trabecular volume (BV/TV; Fig 5B) and relative trabecular surface (BS/BV; Fig 5B) were unaffected. Trabecular number was elevated in the Opa3^L122P^ mice by 13% (Tb.N; Fig 5B; *P*<0.05), and with no change in trabecular thickness (Tb.Th; Fig 5B), trabecular separation was reduced by 19% (Tb.Sp; data not shown; *P*<0.05).

Three-point bending revealed that mechanical strength of the mid-diaphyseal cortex (Ultimate moment) was profoundly compromised in Costeff syndrome mice, being reduced by 65% (Fig 5C; *P*<0.001). This was due to a combination of a 35% reduction on the strength of the calcified tissue per se (ultimate tensile stress; Fig 5C; *P*<0.01) and a 55% reduction in the geometric contribution (second moment of area; Fig 5C; *P*<0.01). Given the clear association between femoral strength and body weight (Fig 5C; R^2^ = 0.9118), body weight-corrected femoral strength was not significantly different in the Opa3^L122P^ model for Costeff syndrome.

*Vertebral structure and strength in Costeff syndrome mice*

As in our microscopic quantification of the marrow compartment in the L3 vertebral body above, the size of the L5 vertebrae in Opa3^L122P^ mice was reduced in all dimensions, vertebral length, and the cross sectional area of the vertebral body being reduced by 24% (Fig 6A; *P*<0.001) and 15% (Fig 6A; *P*<0.05) respectively. The thickness of the cortical bone surrounding the vertebral body was also reduced by 28% (Fig 6A; *P*<0.001).

As in the femora, trabecular structure in the L5 vertebrae was characterized by a denser lattice of smaller trabeculae. This was due to a 38% increase in trabecular number (structure linear density; Fig 6B; *P*<0.01), a 9% increase in relative surface (BS/BV; *P*<0.01), with trabecular thickness and trabecular separation reduced by 5% (Tb.Th; Fig 6B; *P*<0.05) and 25% (Tb.Sp; Fig 6B; *P*<0.01) respectively. The relative preponderance of rod- or plate-like trabeculae (structure modal index), and the connectivity (pattern factor) and orientation of the trabecular lattice (degree of anisotropy) were not significantly affected in Costeff syndrome mice (data not shown).

Despite this evidence of a denser trabecular lattice in Costeff syndrome vertebrae, the load required to crush the L5 vertebral body of Opa3^L122P^ mice (F_max_) was reduced by 35% (Fig 6C; *P*<0.05). Interestingly, given the relationship between F_max_ and cross sectional area (Fig 6C), calculation of maximum stress (σ_max_) revealed that mean σ_max_ in Costeff syndrome mice was 169% of that in W-T mice, though this was not statistically significant (*P*=0.065). Given the weak relationship between σ_max_ and trabecular number (data not shown), normalization of σ_max_ to trabecular number, revealed that any elevation in maximum stress in Costeff syndrome vertebrae was entirely due to the denser trabecular lattice (Fig 6C).

**Discussion**

Costeff syndrome is a mitochondriopathy in which mutations in the mitochondrial membrane protein, Opa3, result in a range in neurological deficits. Mice bearing a missense mutation of Opa3 recapitulate many of the symptoms of this condition (18,24,25) and exhibit a metabolic impairment not previously associated with Costeff syndrome (24). Given the bi-directional relationship between fat and bone (26), we characterized the skeletal phenotype in this model for Costeff syndrome, shedding new light on the role of mitochondria in bone physiology and of the impact of this condition on skeletal integrity.

Our data reveal Opa3 to be expressed in both the developing and adult mouse skeleton and that global mitochondrial dysfunction in Costeff syndrome mice has marked consequences for skeletal growth and homeostasis. In the hindlimb, elevated marrow adiposity is accompanied by reductions in femoral size and tissue strength, resulting in profoundly compromised cortical strength. In contrast, although the compressive strength of the vertebrae is reduced, the trabecular contribution to strength is increased in parallel with the rise in trabecular number, marrow adiposity being slightly reduced. Although we did not analyse the biomechanical properties of the skull, skulls of Costeff syndrome mice showed evidence of reduced longitudinal growth. This was most prominent in the lower mandible in which Opa3 expression was prominently expressed during embryogenesis.

Several mechanisms may account for these changes. Firstly, the reduction in ultimate tensile stress in the femoral cortex implies that the processes of matrix formation and/or mineralization are compromised. The reduced cortical thickness in both femora and vertebrae, when combined with our evidence for Opa3 expression in discrete cells on the endosteal surface suggests impairment of osteoblast function. Indeed, previous reports that osteoblast mitochondria regulate differentiation (10,11) and mineralization (12,13,14) support this proposal.

However, the increased trabecular number in Costeff syndrome mice indicates that mitochondria may play a different role in cancellous bone. Increased trabecular density could result from either elevated osteoblastic activity, or reduced osteoclastic activity. Our evidence seems to favour the former, as Opa3 immunoreactivity is most pronounced on the convex surfaces of the trabeculae, which suggests osteoblast-specific expression. The increased trabecular density not only accounts for the potential increase in σ_max_ in Costeff syndrome vertebrae, but explains why the deficit in mechanical strength of the vertebrae, in which trabecular structure makes a greater contribution, is less pronounced than in the femoral cortex. Indeed, since in compression testing the trabeculae fail as a result of buckling, their strength may be enhanced as a result of a reduction in their unsupported length.

Whilst it may be possible that osteoblast mitochondria exert differential effects in these two types of bone, an alternative explanation is that the weakening of cortical bone in Costeff syndrome mice results from a failure in the maintenance of skeletal integrity. This is supported by our demonstration of marked Opa3 expression in cortical osteocytes.

In addition to these direct effects on bone cell activity, the relationship between cortical strength and body weight, implies a contribution from the more indirect consequences of mitochondrial impairment. In this context, the positive relationship between mechanical load and bone strength (27) may be significant. Loss of mitochondrial function in BAT of Costeff syndrome mice results in profound intra-abdominal leanness and a 60% reduction in body weight (24). This partial unloading may explain the greater impairment in the load-bearing limb bones than in the axial bones. Although the relatively normal appearance of the calvarial bone supports this hypothesis, several additional factors may also contribute to this phenotype.

Given their degree of leanness, Costeff syndrome mice are likely to be hypoleptinemic. While hyperleptinemia is thought to suppress bone formation via a central mechanism (28,29), leptin within the normal range directly promotes osteoblastic activity (30,31). Thus, loss of circulating leptin may contribute to the compromised bone integrity. However, the marked truncal leanness is accompanied by a remarkable rise in tibial marrow adipogenesis. Since marrow adipocytes themselves produce high levels of leptin (32) and direct infusion of leptin into the tibial marrow cavity increases osteoblast surface and reduces marrow adipogenesis (33), the presence of weakened limb bones in the context of a sustained increased marrow adiposity in Costeff syndrome mice implies that this action of leptin is dependent upon mitochondrial activity. Indeed, the importance of mitochondrial activity is emphasized by the combination of increased marrow adiposity and trabecular number in the limb bones of Opa3^L122P^ mice. The parallel increase in these parameters is unusual, as trabecular number is normally inversely related to marrow adiposity (34), especially in the context of dietary manipulation (35).

The site-specific changes in marrow adiposity deserve comment. Like intra-abdominal adipocytes, tibial marrow adipocytes are exquisitely sensitive to growth hormone (GH), ghrelin and leptin exposure, but are unresponsive to IGF-1 and PTH (34,36,37). However, given the profound leanness in Costeff syndrome mice (24), the current study reveals that marrow adipocytes are not only regulated independently of intra-abdominal adipocytes, but are even regulated differentially in the two locations studied. Although the physiological significance of marrow fat is not fully understood, periods of caloric restriction have been reported to elevate marrow adiposity (38), while marrow adiposity is conserved during starvation (39) and in patients with acquired generalized lipodystrophy (40). This situation may reflect the sensitivity of these cells to ghrelin, which promotes adipogenesis rather than lipid storage in marrow adipocytes (33), thereby defending this fat deposit against utilisation (41).

These considerations aside, the profile of fat distribution seen here in Costeff syndrome mice is unusual, even in the context of murine lipodystrophy (42). Therefore comparison with rodent models showing a similar distribution may suggest additional potential mechanisms. For example, several of the phenotypic characteristics of Costeff syndrome mice seen here are replicated in the profoundly GH-deficient dwarf (*dw/dw*) rat. These animals, bearing an unknown mutation, display truncal leanness and hypoleptinemia (43), with elevated marrow adiposity and compromised femoral strength (44). Indeed, other rodent models of GH-deficiency/insensitivity also show reduced mandibular growth and malocclusion (45,46). Direct measurement of episodic GH secretion in Costeff syndrome mice would be technically challenging, but the dramatic reduction in circulating IGF-1 (24) indicates that further analysis of mitochondrial function in the GH-IGF-1 axis of Costeff syndrome mice is now required.

Our study highlights the potential subcellular role of Opa3 in adipocytes and bone mitochondria. A recent report indicates that Opa3 binds to the molecular chaperone, prohibitin (47), which maintains the integrity of the inner mitochondrial membrane (48) and shuttles cardiolipin between the mitochondria and the nucleus (49). Thus, failure of Opa3 to anchor prohibitin to the inner mitochondrial membrane of Costeff syndrome mice would render tissues with high energy demands susceptible to hypoxia and oxidative stress. Our data indicate that this function may be important in the cells regulating bone development and health.

When taken together, we have demonstrated that homozygous Opa3^L122P^ mice display the hallmark characteristics of Costeff syndrome, 3-MGA (24), retinal abnormality, optic atrophy and loss of visual acuity consistent with optic nerve degeneration (18). Other neural deficits include spasticity and extrapyramidal dysfunction and gross neuromuscular defects (18). In addition, the disrupted mitochondrial morphology in Opa3^L122P^ mice (25) results in reduced lifespan, dilated cardiomyopathy (18) and impaired thermogenesis in interscapular BAT resulting in profound intra-abdominal leanness (24). We have now shown that these metabolic disturbances are accompanied by post-natal growth retardation (24) compromised skeletal development and impaired biomechanical integrity. Undergrowth of the lower mandible in Opa3^L122P^ mice, is accompanied in some individuals by cranial asymmetry and incisor malocclusion. Reduced tibial length (24) is accompanied by a marked elevation in tibial marrow adiposity. Reductions in femoral length, cortical diameter and wall thickness, together with weakening of the calcified tissue, result in a markedly compromised cortical strength. Although vertebral body area and wall thickness were reduced in lumbar vertebrae and the total biomechanical strength was reduced, the strength of the calcified tissue was increased in proportion to the elevated trabecular number. Despite these multi-system abnormalities in homozygous Opa3^L122P^ mice, heterozygous mice, at least in relation to their general appearance and the optic and neural defects, are indistinguishable from their wild-type littermate controls (18).

In summary, the current study demonstrates that impaired mitochondrial function in the Opa3^L122P^ model for Costeff syndrome disturbs the functional relationship between adipocytes, osteoblasts and osteoclasts, resulting in compromised mechanical performance of both axial and limb bones. Given that the processes of longitudinal bone growth and skeletal remodelling are remarkably similar in rodents and humans (50), our data have significant implications for individuals with Costeff syndrome. Although evidence has recently emerged of limb dystonia (51,52) and ataxia (52,53), there do not appear to be any extant studies of skeletal development, bone microarchitecture or fracture risk in the identified Costeff syndrome kindreds. Although it is possible that the skeletal impairment in the Opa3^L122P^ mice relates to the specific mutation in this model, Costeff syndrome has been reported in humans with mutations in exon2 of Opa3 (19,23). In the light of our current study, an investigation of bone health in individuals with this condition should now be undertaken. Indeed, when taken together with recent evidence indicating that low circulating mitochondrial DNA is associated with decreased femoral neck BMD in postmenopausal women (54), our study indicates that a wider analysis of the importance of mitochondrial activity to bone health is now necessary.

**Materials and Methods**

*Animals*

The procedures described conformed to the institutional and national ethical guidelines for animal experimentation, including those for genetically modified animals, and were specifically approved by local ethical review. Mice were bred in the JBIOS facility at Cardiff University and housed under conditions of 12h light:12h dark (lights on at 06.00h), ambient temperature (19-23°C), with food (Harlan Teklad Rodent Maintenance Diet containing 4.9% oil and 14.2% protein) and water available *ad libitum*.

To investigate Opa3 mRNA expression in WT mouse head development, heads were collected from CD1 mice at E10.5, E11.5, E14.5 and E18.5, fixed in 4% paraformaldehyde and decalcified in 0.5 M EDTA acid (pH7.6), paraffin-embedded and serially sectioned at 7µm, with sections distributed between 5–10 slides. Radioactive section *in situ* hybridization (ISH) using 35S-UTP radiolabelled riboprobes was carried out as described previously (55). Tibiae were collected from 6-month old male WT mice (C57Bl6) killed by cervical dislocation for subsequent immunohistochemical (IHC) identification of Opa3 distribution.

To investigate the effect of the L122P mutation on cranial development, an additional cohort of whole heads were collected from WT and Opa3^L122P^ mice at E18.5. Heads were fixed in 95% ethanol, stained with Alcian blue and Alizarin red S, cleared by KOH treatment and stored in 100% glycerol prior to whole mount visualization under light microscopy. Additional whole heads were collected from young (P14) and adult (4 month-old) WT and Opa3^L122P^ littermates were killed by either anaesthesia with isoflurane and decapitation, or lethal injection and stored in 4% paraformaldehyde prior to analysis of the structure of the skull, jaw and denture by micro-computed tomography (µ-CT).

The remaining studies were performed on tissue collected from male homozygous Opa3^L122P^ mice and WT littermates killed by decapitation at 30 days of age after being anaesthetized with isofluorane. Right tibiae and femora were excised and cleaned of soft tissue. Tibiae were fixed and decalcified prior to paraffin-embedding and quantification of marrow adiposity. Femora were wrapped in saline soaked gauze and stored at -70°C prior to quantification of trabecular architecture and strength testing. In addition, the spine was exposed and vertebrae L2-L6 excised. L3 and L5 vertebrae were carefully dissected and cleaned of soft tissue. L3 vertebrae were fixed overnight in buffered formal saline and decalcified for 2 weeks prior to quantification of marrow adiposity. L5 vertebrae were wrapped in saline-soaked gauze and stored at -70°C prior to quantification of trabecular architecture and mechanical strength.

*Immunohistochemistry for Opa3*

An IHC approach was developed for visualisation of Opa3 distribution in 7µm sections of paraffin-embedded tissue from WT mice. A rabbit anti-human/mouse/ratOpa3 primary antibody (optimal dilution 1:100 (Supplementary figure 1); Cat # 15638-1-AP; ProteinTech, Chicago Illinois, USA) in combination with the ImmPRESS peroxidase secondary antibody and the BCIP/NBT peroxidase substrate (Cat #s MP-7401 & SK-5400; Vector Laboratories Inc., Burlingame, CA, USA) was used to visualise Opa3 expression, the tissue being counterstained with haematoxylin and eosin. The method was initially developed for use with brown adipose tissue, which showed marked expression surrounding the lipid-containing vesicles, and subsequently applied to tibial sections. No Opa3 immunoreactivity was seen on omission of the primary antibody (Fig 2).

*Microstructural analysis with µ-CT*

*Heads:* Heads of W-T and Opa3L122P mice were scanned with an Explore Locus SP (GE Pre-Clinical Imaging, London, Canada). The heads were immobilised using cotton gauze and scanned to produce 28µm voxel size volumes, using an X-ray tube voltage of 80kVp and a tube current of 80µA. An aluminium filter (0.05mm) was used to adjust the energy distribution of the X-ray source. The heads were characterised by making three-dimensional slice volumes and segmented using automatic threshold functioning. Stacked 2-D scans were rendered into 3D using ImageJ (1.49v for MacIntosh, National Institutes of Health, Bethesda, Maryland, USA) and linear measurements made of skull length, nasal height and length, mandibular height and length as previously described (45), with the length and misalignment of the upper and lower incisors measured using the co-ordinates shown in Figure 7B & C. In addition, external cranial volume was calculated as the volume of a spheroid, using the equation:

4/3 π x cranial length x cranial height x cranial width

using the co-ordinates shown in Figure 7A & B. Similarly, the dome angle was calculated by drawing a line joining the intersection of the interparietal bones and the squamous portion of the occipital bone with the ridge dorsal to the junction of the maxilla with the frontal and lacrymal bones, then determining the position of the perpendicular line giving the highest point to the dorsal surface. Using these co-ordinates (Fig 7B), dome angle (θ; in degrees) was calculated as:

θ = arctan(yz/xy) x (360/2π)

*Femora:* Trabecular microarchitecture was assessed in the distal femora using a high-resolution µ-CT system (Skyscan 1072, Kontich, Belgium) as previously described in rats (35). Femora were thawed immediately before scanning, mounted on the sample presentation stage and orientated by taking a series of single images. Scanning was conducted at 50kV and 210µA, using a resolution of 5.57µm, 8 second exposures, a magnification of 27.34x, a rotation step of 0.45° and a 1mm aluminium filter. After scanning, each femur was re-wrapped in saline-soaked gauze, re-frozen and stored for subsequent strength testing. An area of the secondary spongiosa of the distal femora (1mm^3^ 0.5mm below the center of the epiphyseal plate) was analyzed using the CT analyzer (CT-*An*; <http://www.skyscan.be/products/nrecon.htm>). Trabecular bone was separated from cortical bone within the area of interest by using the freehand drawing tool in CT-*An*.

*Vertebrae:* Trabecular architecture in L5 vertebral bodies was assessed as above, but due to their small size, vertebrae were stabilized with cotton wool inside a 1.5ml Eppendorf tube, the cotton wool being moistened with phosphate-buffered saline to prevent dehydration. Scanning was conducted at using the same parameters as above and the vertebrae re-frozen prior to strength testing. Before analysing the trabecular architecture, reconstructed 3D images were rotated with DataViewer (<http://www.skyscan.be/products/dataviewer.htm>) to achieve a consistent orientation. A 1.002mm transverse section 30 slices (0.176mm) rostral of the centre of the caudal growth plate was analyzed using CT-*An*. The region of interest was restricted to the trabecular bone within the vertebral body. In addition, vertebral size was determined by measuring the distance from the centre of the epiphyseal plates and the thickness of the cortical bone at the most dorsal point of the vertebral body measured.

*Strength testing: a. three point bending in femora*

Mechanical strength of the femoral cortex was quantified by three-point bending as previously described (41), with the lower rollers set at 6.42 and 4.04 mm apart for WT and Opa3^L122P^ femora respectively and the central roller positioned equidistant from the lower rollers over the thinnest part of the mid-diaphyseal region, to give a roughly posterior load direction. Each bone was loaded at a crosshead speed of 2mm/min until failure, with load and displacement data recorded by a Zwick Z050 tensile testing machine fitted with a 1kN load cell (Zwick Testing Machines Ltd., Leominster, Herefordshire, United Kingdom). Ultimate tensile stress was calculated using failure load, morphometric measurements of cortical wall thicknesses and diameters (quantified from the cross-sectional µ-CT image corresponding to the site of fracture as determined by measuring the distance from the end of the femur to the fracture point using a hand-held micrometer) and simple beam theory (41).

*Strength testing: b. compressive strength in vertebrae*

We adapted the method of Moskilde et al (56) to quantify the mechanical strength of the L5 vertebrae. After removing any remaining soft tissue, the neural arch and transverse processes were carefully removed from each vertebra, to leave only the vertebral body. The caudal epiphyseal plate was carefully removed and the sample mounted caudal end down in Araldite on a small metal plate and left for 24h to set. The rostral epiphyseal plate was then removed with a diamond saw, prior to the collection of a 0.8mm section of vertebral body consisting of the trabecular core and cortical border, but without any growth plate or other soft tissue. Compressive strength of these vertebral body slices was tested in a rostro-caudal direction using the Zwick Z050 tensile testing machine. Load-deformation curves were recorded continuously via the Zwick Test Expert software, with maximum stress (σ_max_) calculated by dividing maximum load (F_max_) by the cross sectional area of the vertebral body (as determined by µ-CT).

*Quantification of marrow adiposity*

Marrow adiposity was determined as previously described (33,36,43). In brief, three 7µm anterior-posterior tibial sections and three dorso-ventral vertebral body sections were stained with Toluidene Blue. Digital photomicrographs were analysed with National Institutes of Health (NIH) Image J, to quantify %-adiposity, adipocyte number and the adipocyte size profile.

*Statistics*

Results are expressed as mean ± SEM, and compared by unpaired Student’s t-test (using GraphPad Prism, GraphPad Software Inc., San Diego. CA., USA), with *P* < 0.05 considered significantly different.

**Funding**

This work was supported by the Medical Research Council (Grant number G0500790: PTS; MV), Cardiff School of Biosciences (AEN; EJC; TW), Cardiff University School of Medicine (BAJE) and a ThermoFisher Inspire Award (MV; TW).

**Acknowledgements**

The authors thank Elaine Taylor and Derek Scarborough (Cardiff University) for excellent technical and histological support, Dr Natalie Jones (King’s College London) for scanning of adult mouse heads and the staff of the JBIOS animal facility for their outstanding animal care.

**Conflict of interest statement**

The authors have no conflicts of interest to disclose

**Reprint requests and correspondence should be addressed to:**

Dr Timothy Wells

School of Biosciences

Cardiff University

Museum Avenue

Cardiff, CF10 3AX

United Kingdom

**References**

1. Finsterer,J. (2004) Mitochondriopathies. *Eur. J. Neurol.*, **11**, 163-186.
2. Wallace,D.C,. and Fan,W. (2009) The pathophysiology of mitochondrial disease as modelled in the mouse. *Genes Dev.* **23**, 1714-1736.
3. Wallace,D.C. (2010) Mitochondrial DNA mutations in disease and aging. *Environ. Mol. Mutagen.* **51**, 440-450.
4. Stambough,J.L., Brighton,C.T., Iannotti,J.P. and Storey,B.T. (1984) Characterization of growth plate mitochondria*. J. Orthop. Res.* **2**, 235-246.
5. Brighton,C.T. and Hunt,R.M. (1978) The role of mitochondria in growth plate calcification as demonstrated in a rachitic model. *J. Bone Joint Surg. Am.* **60**, 630-639.
6. Rajpurohit,R., Mansfield,K., Ohyama,K., Ewert,D. and Shapiro,I.M. (1999) Chondrocyte death is linked to development of a mitochondrial membrane permeability transition in the growth plate. *J. Cell. Physiol.* **179**, 287-296.
7. Miedlich,S.U., Zalutskaya,A., Zhu,E.D.and Demay,M.B. (2010) Phosphate-induced apoptosis of hypertrophic chondrocytes is associated with a decrease in mitochondrial membrane potential and is dependent upon Erk1/2 phosphorylation. *J. Biol. Chem.* **285**, 18270-18275.
8. Kassem,M., Rungby,J., Mosekilde,L. and Eriksen,E.F. (1992) Ultrastructure of human osteoblasts and associated matrix in culture. *APMIS*. **100**, 490-497.
9. Brown,D. and Breton,S. (1996) Mitochondria-rich, protein-secreting epithelial cells. *J. Exp. Biol.* **199**, 2345-2358.
10. An,J.H., Yang,J.Y., Ahn,B.Y., Cho,S.W., Jung,J.Y., Cho,H.Y., Cho,Y.M., Kim,S.W., Park,K.S., Kim,S.Y. *et al*. (2010) Enhanced mitochondrial biogenesis contributes to Wnt induced osteoblastic differentiation of C3H10T1/2 cells. Bone. **47**, 140-150.
11. Chen,C.T., Shih,Y.R., Kuo,Y.K., Lee,O.K. and Wei,Y.H. (2008) Coordinated changes of mitochondrial biogenesis and antioxidant enzymes during osteogenic differentiation of human mesenchymal stem cells. *Stem Cells.* **26**, 960-968.
12. Plachot,J.J., Thil,C.L., Enault,G., Halpern,S., Cournot-Witmer,G. and Balsan,S. (1986) Mitochondrial calcium and bone mineralization in the rat fetus. *Bone Miner.* **1**, 157-166.
13. Manston,J. and Katchburian,E. (1984) Demonstration of mitochondrial mineral deposits in osteoblasts after anhydrous fixation and processing. *J. Microsc.* **134**, 177-182.
14. Troyan,M.B., Gilman,V.R. and Gay,C.V. (1997) Mitochondrial membrane potential changes in osteoblasts treated with parathyroid hormone and estradiol. *Exp. Cell. Res.* **233**, 274-280.
15. Zaman,G., Saxon,L.K., Sunters,A., Hilton,H., Underhill,P., Williams,D., Price,J.S., Lanyon,L.E. (2010) Loading-related regulation of gene expression in bone in the contexts of estrogen deficiency, lack of estrogen receptor alpha and disuse. *Bone*. **46**, 628-642.
16. Yamamoto,N., Fukuda,K., Matsushita,T., Matsukawa,M., Hara,F. and Hamanishi,C. (2005) Cyclic tensile stretch stimulates the release of reactive oxygen species from osteoblastic-like cells. *Calcif. Tissue Int.* **76**, 433-438.
17. Ishii,K.A., Fumoto,T., Iwai,K., Takeshita,S., Ito,M., Shimohata,N., Aburatani,H., Taketani,S., Lelliott,C.J., Vidal-Puig,A. *et al*. (2009) Coordination of PGC-1beta and iron uptake in mitochondrial biogenesis and osteoclast activation. *Nat. Med.* **15**, 259-266.
18. Davies,V.J., Powell,K.A., White,K.E., Yip,W., Hogan,V., Hollins,A.J, Davies,J.R., Piechota,M., Brownstein,D.G., Moat,S.J. *et al*. (2008) A missense mutation in the murine *Opa3* gene models human Costeff syndrome. *Brain*. **131**, 368-80.
19. Anikster,Y., Kleta,R., Shaag,A., Gahl,W.A. and Elpeleg,O. (2001) Type III 3-methyglutaconic aciduria (optic atrophy plus syndrome, or Costeff optic atrophy syndrome): identification of the *OPA3* gene and its founder mutation in Iraqi Jews. *Am. J. Hum. Genet.* **69**, 1218-1224.
20. Ryu,S.W., Jeong,H.J., Choi,M., Karbowski,M. and Choi,C. (2010) Optic atrophy 3 as a protein of the mitochondrial outer membrane induces mitochondrial fragmentation. *Cell. Mol. Life Sci.* **67**, 2839-2850.
21. Pei,W., Kratz,L.E., Bernardini,I., Sood,R., Yokogawa,T., Dorward,H., Ciccone,C., Kelley,R.I., Anikster,Y., Burgess,H.A. *et al*. (2010) A model of Costeff Syndrome reveals metabolic and protective functions of mitochondrial OPA3. *Development*. **137**, 2587-2596.
22. Wortmann,S.B., Kluijtmans,L.A., Engelke,U.F., Wevers,R.A. and Morava,E. (2012) The 3-methylglutaconic acidurias: what’s new? *J. Inherit. Metab. Dis*. **35**, 13-22.
23. Kleta,R., Skovby,F., Christensen,E., Rosenberg,T., Gahl,W.A. and Anikster,Y. (2002) 3-Methylglutaconic aciduria type III in a non-Iraqi-Jewish kindred: clinical and molecular findings. *Mol. Genet. Metab.* **76**, 201-206.
24. Wells,T., Davies,J.R., Guschina,I.A., Ball,D.J., Davies,J.S., Davies,V.J., Evans,B.A. and Votruba,M. (2012) Opa3, a novel regulator of mitochondrial function, controls thermogenesis and abdominal fat mass in a mouse model for Costeff syndrome. *Hum. Mol. Genet*. **21**, 4836-4844.
25. Powell,K.A., Davies,J.R., Taylor,E., Wride,M.A. and Votruba,M. (2011) Mitochondrial localization and ocular expression of mutant Opa3 in a mouse model of 3-methylglutaconicaciduria type III. *Invest. Ophthalmol. Vis. Sci*. **52**, 4369-4380.
26. Leiben,L., Callewaert,F. and Bouillon,R. (2009) Bone and metabolism: a complex crosstalk. *Horm. Res*. **71** Suppl 1, 134-138.
27. Martin,R.B. (1991) Determinants of the mechanical properties of bones. *J. Biomech.* **24** Suppl 1, 79-88.
28. Ducy,P., Amling,M., Takeda,S., Priemel,M., Schilling,A.F., Beil,F.T., Shen,J., Vinson,C., Rueger,J.M. and Karsenty,G. (2000) Leptin inhibits bone formation through a hypothalamic relay: a central control of bone mass. *Cell*. **100**, 197-207.
29. Hamrick,M.W. and Ferrari,S.L. (2008) Leptin and the sympathetic connection of fat to bone. *Osteoporos. Int*. **19**, 905-12.
30. Thomas,T., Gori,F., Khosla,S., Jensen,M.D., Burguera,B. and Riggs,B.L. (1999) Leptin acts on human marrow stromal cells to enhance differentiation to osteoblasts and to inhibit differentiation to adipocytes. *Endocrinology*. **140**, 1630-1638.
31. Lee,Y.J., Park,J.H., Ju,S.K., You,K.H., Ko,J.S. and Kim,H.M. (2002) Leptin receptor isoform expression in rat osteoblasts and their functional analysis. *FEBS Lett.* **528**, 43-47.
32. Laharrague,P., Larrouy,D., Fontanilles,A.-M., Truel,N., Campfield,A., Tenenbaum,R., Galitzky,J., Corberand,J.X. Pénicaud,L. and Casteilla,L. (1998) High expression of leptin by human bone marrow adipocytes in primary culture. *FASEB J*. **12**, 747-752.
33. Thompson,N.M., Gill,D.A., Davies,R., Loveridge,N., Houston,P.A., Robinson,I.C. and Wells,T. (2004) Ghrelin and des-octanoyl ghrelin promote adipogenesis directly in vivo by a mechanism independent of the type 1a growth hormone secretagogue receptor. Endocrinology. **145**, 234-242.
34. Abdallah,B.M. and Kassem,M. (2012) New factors controlling the balance between osteoblastogenesis and adipogenesis. *Bone* **50**, 540-545.
35. Pando,R., Masarwi,M., Shtaif,B. Idelevich,A., Monsonego-Ornan,E., Shahar,R., Phillip,M. and Gat-Yablonski,G. (2014) Bone quality is affected by food restriction and by nutrition-induced catch-up growth. *J. Endocrinol.* **223**, 227-239.
36. Gevers,E.F., Loveridge,N. and Robinson,I.C. (2002) Bone marrow adipocytes: a neglected target tissue for growth hormone. *Endocrinology*. **143**, 4065-4073.
37. Evans,B.A., Bull,M.J., Kench,R.C., Fox,R.E., Morgan,L.D., Stevenson,A.E., Gevers,E.F., Perry,M.J. and Wells,T. (2011) The influence of leptin on trabecular architecture and marrow adiposity in GH-deficient rats. *J. Endocrinol.* **208**, 69-79.
38. Devlin,M.J., Cloutier,A.M., Thomas,N.A., Panus,D.A., Lotinun,S., Pinz,I., Baron,R., Rosen,C.J. and Bouxsein,M.L. (2010) Caloric restriction leads to high marrow adiposity and low bone mass in growing mice. *J. Bone Miner. Res*. **25**, 2078-2088.
39. Bathija,A., Davis,S. and Trubowitz,S. (1979) Bone marrow adipose tissue: a response to acute starvation. *Am. J. Hematol*. **6**, 191-8.
40. Misra,A. and Garg,A. (2003) Clinical features and metabolic derangements in acquired generalized lipodystrophy: case reports and review of the literature. *Medicine (Baltimore).* **82**, 129-46.
41. Wells,T. (2009) Ghrelin – defender of fat. *Prog. Lipid. Res.* **48**, 257-74.
42. Reitman,M,L. (2002) Metabolic lessons from genetically lean mice. *Annu. Rev. Nutr.* **22**, 459-82.
43. Davies,J.S., Gevers,E.F., Stevenson,A.E., Coschigano,K.T., El-Kasti,M.M., Bull,M.J., Elford,C., Evans,B.A., Kopchick,J.J. and Wells,T. (2007) Adiposity profile in the dwarf rat: an unusually lean model of profound growth hormone deficiency. *Am. J. Physiol. Endocrinol. Metab*. **292**, E1483-E1494.
44. Stevenson,A.E., Evans,B.A.J., Gevers,E.F., Elford,C., McLeod,R.W.J., Perry,M.J., El-Kasti,M.M., Coschigano,K.T., Kopchick,J.J., Evans,S.L. *et al*. (2009) Does adiposity status influence femoral cortical strength in rodent models of growth hormone deficiency? *Am. J. Physiol. Endocrinol. Metab*. **296**, E147-E156.
45. Ramirez-Yañez,G.O., Smid,J.R., Young,W.G. and Waters,M.J. (2005) Influence of growth hormone on the craniofacial complex of transgenic mice. *Eur. J. Orthod.* **27**, 494-500.
46. Kuramoto,K., Tahara,S., Sasaki,T., Matsumoto,S., Kaneko,T., Kondo,H., Yanabe,M., Takegi,S. and Shinkai,T. (2010) Spontaneous dwarf rat: a novel model for aging research. *Geriatr. Gerontol. Int.* **10**, 94-101.
47. Um,J.Y., Sripathi,S.R., Lamoke,F., McDonough,R., Bruetle,S., Moser,T., DeBenedetti,R., He,W., Bartoli,M. et al. (2012) Mitochondria-nucleus communication by shuttle proteome in the RPE under oxidative stress (Abstract). In: *Proceedings of AVRO2012*, Fort Lauderdale, Florida, USA, **1494**, A547.
48. Osman,C., Merkwirth,C. and Langer,T. (2009) Prohibitins and the functional compartmentalization of mitochondrial membranes *J. Cell Sci.* **122**, 3823-3830.
49. Sripathi,S.R., He,W., Atkinson,C.L., Smith,J.J., Liu,Z., Elledge,B.M. and Jahng,W.J. Mitochondrial-nuclear communication by prohibitin shuttling under oxidative stress. *Biochemistry*. **50**, 8342-51.
50. Frost,H.M. and Jee,W.S. (1992) On the rat model of human osteopenias and osteoporoses. *Bone Miner*. **18**, 227-236.
51. Grau,T., Burbulla,L.F., Engl,G., Delettre,C., Delprat,B., Oexle,K., Leo-Kottler,B., Roscioli,T., Krüger,R., Rapaport,D. *et al*. (2013) A novel heterozygous *OPA3* mutation located in the mitochondrial target sequence results in altered steady-state levels and fragmented mitochondrial network.  *J. Med. Genet*. **50**, 848-858.
52. Arif,B., Kumar,K.R., Seibler,P., Vulinovic,F., Fatima,A., Winkler,S., Nürnberg,G., Thiele,H., Nürnberg,P., Jamil,A.Z. *et al*. (2013) A novel *OPA3* mutation revealed by exome sequencing: an example of reverse phenotyping. *JAMA Neurol*. **70**, 783-787.
53. Yahalom,G., Anikster,A., Huna-Baron,R., Hohhmann,C., Blumkin,L., Lev,D., Tsabari,R., Nitsan,Z., Lerman,S.F., Bin-Zeev,B. *et al*. (2014) Costeff syndrome: clinical features and natural history. *J. Neurol*. **261**, 2275-2282.
54. Kim,J.H. and Lee,D.C. (2012) Low mitochondrial DNA copy number in peripheral blood is associated with femoral neck bone mineral density in postmenopausal women. *J. Rheumatol*. **39**, 1465-72.
55. Ohazama,A., Johnson,E.B., Ota,M.S., Choi,H.Y., Porntaveetus,T., Oommem,S., Itoh,N., Eto,K., Gritti-Linde,A., Herz,J. *et al*. (2008) Lrp4 modulates extracellular integration of cell signaling pathways in development. *PLoS One.* **3**, e4092.
56. Moskilde,L., Danielsen,C.C. and Knudsen,U.B. (1993) The effect of aging and ovariectomy on the vertebral bone mass and biomechanical properties of mature rats. *Bone*. **14**, 1-6.

**Figure legends**

**Figure 1: *Opa3* mRNA expression in the developing mouse head.** Radioactive *in situ* hybridisation of *Opa3* mRNA expression in E10.5 (A) E11.5 (B) E14.5 (C) and E18.5 heads of male WT mice. Submandibular salivary glands (arrow in Ci), developing mandibular bone surrounding Meckel’s cartilage (short arrows in Cii & iii), lower incisor (arrow in Civ), upper molar (arrow in Cv), lower molar (short arrow in Cv), upper incisors (arrow in Cvi), sensory layer of retina (short arrow in Cvii) at E14.5. Hair follicles (arrows in Di), brain (short arrow in Dii) skull bone (arrow in Dii) and upper molar tooth germ (arrow in Diii) at E18.5. Ameloblasts (short arrows in Div-vii) and odontoblasts (arrows in Div-vii) of anterior incisors (Div & vi) and posterior part of incisors (Dv & vii) at E18.5.

**Figure 2: Immunolocalisation of Opa3 in mouse tibiae.** Immunohistochemical visualisation of Opa3 expression in 6-month old WT tibiae with (A,C,E,G,I,K,M,O) and without (B,D,F,H,J,L,N) application of the primary rabbit anti-mouse Opa3 antibody, using the ImmPRESS peroxidase secondary antibody in conjunction with BCIP/NBT peroxidase substrate to produce indigo labeling, all sections counterstained with haematoxylin and eosin. The immunoreactivity observed under low power (A) was located in osteoblasts on the endosteal (black arrowheads) and trabecular (grey arrowheads) (C), surfaces and on the surface of the cortical periostium (white arrowheads; E) and synovial cartilage (light grey arrowheads; G). In addition, Opa3 immunoreactivity was observed in cortical osteocytes (black arrows; E), synovial chondrocytes (dark grey arrows; G), germinal (g), proliferative (p) and hypertrophic (h) growth plate chondrocytes (I,K), mesenchymal stem cell rosettes (white arrows) in the mid-diaphyseal marrow (M) and adipocytes in the proximal (*; I,K) and distal (v;O) marrow, (Scale bars: 300µm (A,B), 50µm (C,D,I,J,M,N,O) and 20µm (E,F,G,H,K,L).

**Table 1: Skull morphometry in 14-day old WT and Opa3^L122P^ littermate mice.** Values shown are mean ± SEM (n=9 (WT; 4 males/5 females) and 5 (Opa3^L122P^; 3 males/2 females)), with statistical comparisons performed with unpaired Student’s t-test (*P<0.05; **P<0.01; ***P<0.001).

**Figure 3: Opa3^L122P^ mice show abnormal skull formation.** 3D reconstructions of µ-CT scans showing dorsal, lateral and frontal aspects of juvenile (14-day old) and adult (4-month old) male WT and Opa3^L122P^ littermate mouse skulls (scale bar 2mm).

**Figure 4: Opa3^L122P^ mice show site-specific dysregulation of marrow adiposity.** Digital analysis of photomicrographs (inset; (adipocytes: black arrows); scale bar 20µm (tibial images) 50µm (vertebral images)) to quantify marrow adiposity, adipocyte number, adipocyte size and size distribution in tibiae (A) and L3 vertebrae (B) from 30-day old male WT (n=6 (tibiae) & 5 (vertebrae)) and homozygous Opa3^L122P^ littermate mice (n=6 (tibiae) & 3 (vertebrae)). Data presented are mean ± SEM, with statistical analysis performed by unpaired Student’s *t* test; a *P*<0.05 vs WT males.

**Figure 5: Opa3^L122P^ mice show compromised femoral strength**. Quantification of the geometry (A) trabecular architecture (B) and mechanical strength of the cortex (C) of femora from 30-day old male WT (n=5) and homozygous Opa3^L122P^ littermate mice (n=5). Data presented are mean ± SEM, with statistical analysis performed by unpaired Student’s *t* test; a *P*<0.05; aa *P* <0.01, aaa *P* <0.001 vs WT males.

**Figure 6: Opa3^L122P^ mice show compromised vertebral strength.** Quantification of the geometry (A) trabecular architecture (B) and mechanical strength (C) of L5 lumbar vertebrae from 30-day old male WT (n=5) and homozygous Opa3^L122P^ littermate mice (n=5). Data presented are mean ± SEM, with statistical analysis performed by unpaired Student’s *t* test; a *P*<0.05; aa *P* <0.01, aaa *P* <0.001 vs WT males.

**Figure 7: Quantification of skull morphology in WT and Opa3^L122P^ mice.** Linear measurements were made of skull length (a-b); cranial length (a-c); cranial height (d-e); cranial width (f-g); cranial dome angle (θ; calculated from the xyz co-ordinates as described in the text); nasal height (h-i); nasal length (i-b), mandibular height (j-k); mandibular length (l-m); upper incisor length (n-o); lower incisor length (p-q); incisor misalignment (r-s) on µ-CT reconstructions showing dorsal (A) right ventral (B), and frontal (C) aspects (scale bar = 2mm).

**Abbreviations**

3-MGA Type III methylglutaconic aciduria

aa Amino acid

BCIP/NBT 5-bromo-4-chloro-3-indolyl phosphate/nitroblue terazolium

BS Bone surface (trabecular)

BMD Bone mineral density

BV Bone volume (trabecular)

F_max_ Maximum load required

GH Growth hormone

IGF-1 Insulin-like growth factor-1

IHC Immunohistochemistry

MSC Mesenchymal stem cell

PTH Parathyroid hormone

Tb.N Trabecular number

Tb.Sp Trabecular separation

Tb.Th Trabecular thickness

TV Total sample volume

µ-CT Micro-computed tomography

σ_max_ Maximum stress required

Navein A *et al* (2016); Table 1

**Table 1: Skull morphometry in 14-day old WT and Opa3^L122P^ littermates**

|  | **WT**  (n=9) | **Opa3^L122P^**  (n=5) |
| --- | --- | --- |
| Skull Length (mm) | 17.43 ± 0.21 | 15.76 ± 0.29 *** |
| External cranial volume (mm^3^) | 1892.37 ± 93.82 | 1644.25 ±104.83 |
| Cranial dome angle (°) | 25.1 ± 1.5 | 23.9 ± 1.9 |
| Nasal height (mm) | 3.88 ± 0.06 | 3.56 ± 0.12 * |
| Nasal length (mm) | 4.69 ± 0.14 | 4.54 ± 0.25 |
| Mandibular height (mm) | 3.38 ± 0.08 | 3.00 ± 0.06 ** |
| Mandibular length (mm) | 6.06 ± 0.09 | 5.23 ± 0.11 *** |
| Upper incisor length (mm) | 1.67 ± 0.10 | 1.45 ± 0.04 |
| Lower incisor length (mm) | 2.36 ± 0.08 | 2.04 ± 0.09 * |
| Incisor misalignment (mm) | 0.03 ± 0.01 | 0.16 ± 0.06 * |

Values shown are mean ± SEM (n=9 (WT; 4 males/5 females) and 5 (Opa3^L122P^; 3 males/2 females)), with statistical comparisons performed with unpaired Student’s t-test (*P<0.05; **P<0.01; ***P<0.001).
